# Pseudoaneurysm in internal maxillary artery after gunshot wound: Critical review and case report

**DOI:** 10.4317/jced.54849

**Published:** 2018-07-01

**Authors:** Bruno Neres, Eugênia Figueiredo, Carolina Aires, Emerson Nogueira, Emanuel Andrade

**Affiliations:** 1 DDS - Oral and Maxillofacial Department, Dental School of Pernambuco, University of Pernambuco, Camaragibe, Pernambuco, Brazil; 2 DDS, MSc - Oral and Maxillofacial Department, Dental School of Pernambuco, University of Pernambuco, Camaragibe, Pernambuco, Brazil; 3 DDS, MSc, PhD, Professor of the Oral and Maxillofacial Department, Dental School of Pernambuco, University of Pernambuco, Camaragibe, Pernambuco, Brazil

## Abstract

**Introduction:**

Pseudoaneurysm is a vascular injury typically caused by rupture of arteries with extravasation of blood. The involvement of this entity in facial arteries after firearm aggression is extremely rare, and they need treatment as early as possible, thus avoiding irreversible damage to patients.

**Study design:**

A 40-year-old male victim of gunshot attack with an entrance orifice in the right posterior cervical region with ascending trajectory, lodging in the ipsilateral zygomatic-orbitary complex, which was submitted to removal of the bone fragments and the bullet. In the intraoperative period, the patient developed profuse hemorrhage and, after complementary examinations, he was diagnosed with pseudoaneurysm of the internal maxillary artery, which was treated by selective endovascular embolization.

**Results:**

The patient was hemodynamically stable, with no complaints and was discharged after 48 hours, without postoperative bleeding recurrences. He had no more complications after 8 months of follow-up.

**Conclusions:**

The main forms of treatment and diagnosis of vascular lesions are reviewed, and embolization is demonstrated as a technically safe procedure with few complications.

** Key words:**Gunshot wound, pseudoaneurysm, maxillary artery, therapeutic embolization.

## Introduction

Pseudoaneurysm (PA) is a vascular injury typically caused by rupture of the arteries with extravasation of blood. The compressed perivascular tissue forms the wall of the aneurysmal sac and develops in the mass with active pulsation. Pseudoaneurysm differs from true aneurysm in that the latter involves abnormal expansion of the arteries due to the weakening of vessel walls with blood accumulating between the arterial wall layer (1).

The traumatic involvement of PAs in the internal maxillary arteries is rare because of the anatomical location and protection of the surrounding tissues, although the other branches of the external carotid artery may be more commonly involved in complications and concomitant vascular lesions. Although uncommon, traumatic PAs of the internal maxillary artery are well-recognized complications in facial trauma. The penetrating and blunt trauma is the most common cause (2).

The internal maxillary artery arises behind the neck of the condylar process of the mandible and is well protected by the maxilla. A traumatic injury can result in deep bleeding and shock due to its deep location of difficult control only by direct compression (3). There are several ways to deal with this type of bleeding, including surgical intervention, embolization and nasal packing (4).

The objective of this study is to report an unusual case of pseudoaneurysm of the internal maxillary artery in a victim of a gunshot wound treated by catheter embolization using the micro springs technique, as well as to make a review on the subject.

## Case Report

A 40-year-old male patient came to the hospital emergency after a gunshot lesion in the cervical region. He was conscious, hemodynamically stable, and without signs of active bleeding or cervical spine injuries. Physical examination showed significant edema in the region of the mandibular angle, trismus, restriction of mandibular movements, absence of rhinorrhea or epistaxis, and soft tissue injury compatible with the bullet entrance orifice in the right posterior cervical region without clinical signs of exit bullet orifice.

**Figure 1:**
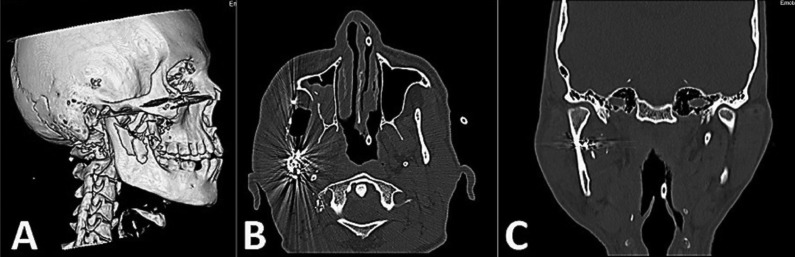
A three-dimensional projection of the CT scan. 2 e 3) CT of the facial bones demonstrates numerous fractures along the bullet trajectory.

**Figure 2:**
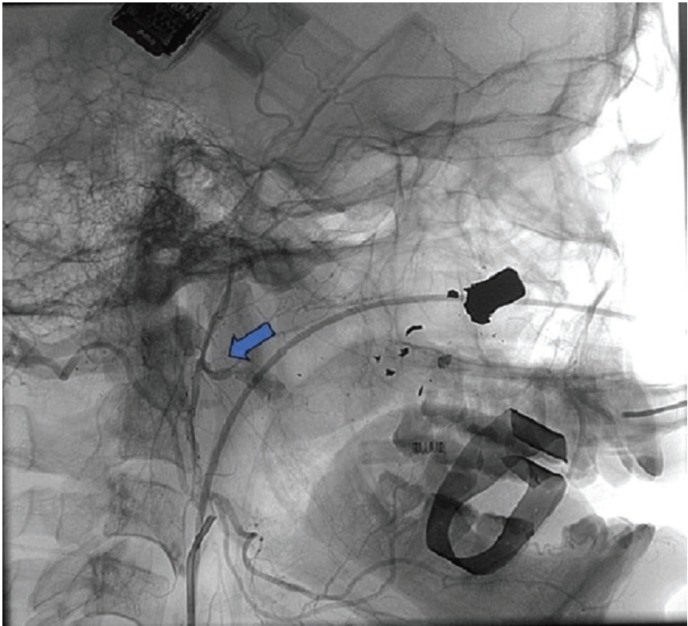
Angiography showing pseudoaneurysm of the right internal maxillary artery (blue arrow).

**Figure 3:**
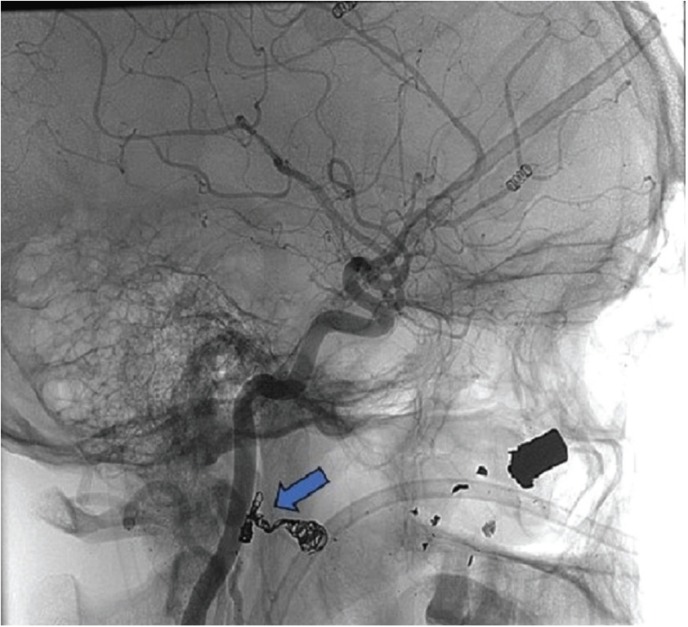
Angiography showing successful occlusion of the pseudoaneurysm after embolization (blue arrow).

Computed tomography showed a comminuted fracture of the coronary and mandibular right ascending branches associated with ipsilateral zygomatic-orbital fracture (Fig. 1) and the presence of artifacts compatible with the firearm projectile, suggesting an upward trajectory toward the face (Figs. 2,3).

After physical and imaging evaluation, vascular surgery and neurosurgery teams opted for conservative treatment. However, the maxillofacial surgery team indicated surgical removal of the bone fragments due to the restrictions of the mandibular movements and removal of the fragments of the projectile due to discomfort and superficialisation in the genic region.

On the third day after trauma, under general anesthesia, removal of the bone fragments was initiated by intraoral access in the ascending ramus of the mandible, which evolved intraoperatively with an intense arterial bleeding, incompatible with the surgical procedure. Local compression maneuvers were performed using compresses, attempts to pinch with instruments after local exploration and the use of hemostatics, but they were not enough to contain the bleeding. After failure, it was decided to submit the patient to angiography of the external carotid artery.

The examination was performed by percutaneous puncture of the right femoral artery and selective catheterization of the external carotid artery and internal maxillary artery, which verified the presence of an PA (Fig. 2) with indication of emergency embolization procedure. Through the catheter, the embolization was performed from the installation of 02 micro-platinum springs until the complete arterial occlusion and consequent end of the blood flow of the PA (Fig. 3). The selective angiography of the left internal maxillary artery was then performed in different projections to rule out another possible source of bleeding and the possibility of compensatory revascularization.

The patient was transferred and remained under observation for 12 hours in the intensive care unit. A new angiography was performed after 24 hours for control, confirming the complete resolution of the PA. The patient was submitted to a second surgery 72 hours after the hemorrhagic episode when a large part of the bone fragments were removed by intraoral access. Also the palpable and superficial portion of the projectile located in the genic region by infraorbital access was removed.

The patient was hemodynamically stable, with no complaints and was discharged after 48 hours, without postoperative bleeding recurrences. He had no more complications after 8 months of follow-up.

## Discussion

Pseudoaneurysms (PAs), or traumatic aneurysms, are different from true aneurysms because they involve rupture of the internal layers of an artery after blunting, penetrating or even surgical trauma. They may also occur in interruptions of all three vascular layers if the original hematoma is buffered by the surrounding soft tissues. In a penetrating trauma, as reported in this case, the usual mechanism is high velocity maxillofacial trauma, in which fragments of bone or projectile lead to arterial laceration (5,6). The progressive increase of the lesion can lead to several complications, including rupture of the aneurysm and hemorrhage, compression of adjacent nerves, or release of embolic thrombi (6).

The rarity of occurrence of PAs after firearm injuries is evidenced by the few cases described in the literature. This demonstrates the lack of data on this topic (5-9). A search in the PubMed database using the terms “pseudoaneurysm”, “maxillary artery” and “gunshot” revealed only 5 available articles described ( Table 1).

**Table 1:**
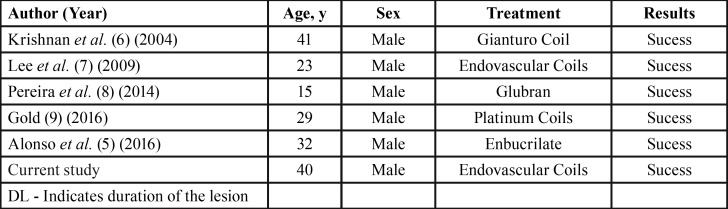
Reported cases of the Formation of Pseudoaneurysms After Gunshot Wound in internal maxillary artery.

After analysis of the literature, it was observed that all the patients described were male, with ages varying from 15 to 41. This data was aligned with the case described here.

Several factors may contribute to the development of PA. The most common causes are accidents or iatrogenic injury and infection associated with the arterial wall (10). Most of the papers describe the trauma (6,11) as the most common cause of PA in the head and neck region. Among traumatic etiologies, penetrating lesions in the external carotid artery to its deeper branches and trauma to the superficial temporal artery are the most commonly described (12).

Most of the PAs of the internal maxillary artery are consequences of penetrating and forceful trauma, and may occur as complications in temporo-mandibular joint surgery, orthognathic surgery, mandibular condyle fractures, as well as the consequence of excessive effort by trumpeters and in the disease of Von Willebrand (3).

The branches of the internal maxillary artery are also vulnerable to lesions at different levels of their anatomical location, either by trauma or surgical intervention. An injury to an arterial wall with maintenance of blood flow produces a hematoma at the boundaries of the surrounding soft tissues. The size of the hematoma is limited by the balance between the pressure inside the vessel and the pressure within the hematoma itself. In a few days, this hematoma begins to liquefy in the center, while producing a new layer of connective tissue along the outer wall. With the progression of central liquefaction, The PA evolves as it pulsates and expands progressively. This may cause pressure on the surrounding tissue, rupture causing severe hemorrhage or embolic thrombi release (13).

The diagnosis of PA is strongly suggested by the patient’s history and physical examination. Patients typically report a pulsatile mass in an area that has recently been traumatized (13,14). In our report, the patient did not show the pulsation of the lesion, probably because the PA was related to a deeper artery. The differential diagnosis includes an arteriovenous fistula, which often has a similar history, but is usually associated with a continuous sound (14). Other lesions considered in the differential diagnosis are tumor, hematoma, abscess or lipoma, which do not present a pulsation.

Angiography is a radiological technique that describes the pathway that feeds the lesion and locates the exact site of bleeding, thus allowing precise treatment (10). In fact, it is the main test used in cases of hemorrhage after injury by gunshot. In the present case, angiography was performed with selective catheterization of the external carotid artery and right internal maxillary artery, which revealed an PA irrigated by this vessel. To stop bleeding caused by PA formation, several authors recommend complete excision of the lesion and ligation of the proximal and distal portions of the artery (12). Occasionally, it may be necessary to ligate the external carotid artery as well as its minor branches, but this is usually hampered by the retraction of small blood vessels after injury. Unfortunately, the effect of an arterial ligature is variable because of collateral circulation and arterial communication with the internal carotid system (12). Other forms of treatment include selective arterial embolization (10,14) and prolonged compression.

Embolization has been widely used in the treatment of vascular lesions and bleeding in the head and neck. This procedure was first described by Dawbom who injected paraffin and Vaseline to block blood flow in a facial sarcoma in 1904. The goal of embolization in cases of PA is not to block blood vessels near the lesion as this would serve to stimulate the growth of a collateral network of blood supply, but rather to deposit a substance in the aneurysm network (12). This is a less invasive method than traditional surgical techniques, it closes the vessel and stops bleeding, thus being a definitive treatment. Selective embolization is best performed with a catheter, which is introduced through the femoral artery to the external carotid artery and its branches. This technique was used in this case.

The most commonly used permanent embolization materials for embolization of vascular lesions in the head and neck are polyvinyl alcohol (PVA), micro springs and liquid adhesives. Polyvinyl alcohol has been widely used for the treatment of idiopathic epistaxis or epistaxis caused by preoperative tumor embolization. The disadvantage of PVA is recurrent PA bleeding after embolization. Sometimes it can be difficult to place the particles in the affected artery due to blockage of the lumen of the microcatheter by the PVA itself. Micro springs, such as platinum springs or removable Guglielmi springs, have been successfully used in the treatment of intracranial aneurysms and arteriovenous fistulas (10,11). The detachable springs have the advantage of being very flexible, having elastic memory and can be placed precisely in the affected artery. Amongst the 5 cases found in the review, the springs were the most used (3 cases), followed by surgical glues (2 cases), where all cases were successfully treated. In addition, in the mentioned clinical case, success was achieved with the use of the endovascular spring.

The great majority of authors adhered to the non-surgical treatment of vascular lesions of the internal maxillary artery. Yin (15) reported the difficulty in achieving adequate hemostasis with multiple ligatures and attributed this to the fact that the internal maxillary artery has a large supply network on the contralateral side and its connection with the internal carotid artery.

Surgical exploration and hemostasis are usually performed as a last resort if embolization procedures are not satisfactory. Percutaneous catheterization embolization is a known, safe, fast, and effective technique for the treatment of these PAs. With this technique, it is possible to reach damaged vessels that are difficult to approach surgically. The technique allows immediate verification and efficacy of treatment by post-embolization angiography.

Excision of an AP is usually the last option in cases where embolization has been ineffective. The contraindications are similar to those of compression therapy. The PAs with signs of local infection are better treated surgically, but this depends on the location of the aneurysm and is easily accessible through open removal (10).

If left untreated, pseudoaneurysm tends to become fatal due to frequent bleeding or thromboembolism. Clinical signs of PA of the internal maxillary artery often arise several weeks or months after trauma. Unlike most cases reported in the literature, this patient presented an almost immediate post-traumatic vascular malformation in his infra-temporal fossa, which was confirmed by angiographic studies.

The treatment of pseudoaneurysm aims to control the proximal and distal arterial blood flow of damaged vessels. Endovascular treatment is the best method because of the anatomical location of the PA of the internal maxillary artery (6). Using this technique, it is possible to reach the lesion without damaging adjacent structures, in addition to allowing the verification of immediate treatment results.

## Conclusions

Pseudoaneurysms of the head and neck region are rare and there are few reports in the literature about gunshot wounds. It was observed that the use of embolization by catheterization is a less invasive, efficient and safe technique in the treatment of these complications, thus avoiding invasive methods of surgical interventions in this anatomical space.
